# Pain sensitivity and shoulder function among breast cancer survivors compared to matched controls: a case-control study

**DOI:** 10.1007/s11764-021-00995-y

**Published:** 2021-01-26

**Authors:** G. H. F. Rasmussen, P. Madeleine, M. Arroyo-Morales, M. Voigt, M. Kristiansen

**Affiliations:** 1 Sport Sciences - Performance and Technology, Department of Health Science and Technology, Aalborg University, Aalborg, Denmark; 2 Department of Physical Therapy, Faculty of Health Sciences, Sport and Health Research Institute, Biohealth Research Institute Granada, University of Granada, Granada, Spain

**Keywords:** Breast cancer survivors, Movement-evoked pain, Muscle strength, Range of motion

## Abstract

**Objective:**

Persistent pain and loss of shoulder function are common adverse effects to breast cancer treatment, but the extent of these issues in comparison with healthy controls is unclear for survivors beyond 1.5 years after treatment. The purpose of this study was to benchmark differences in pressure pain thresholds (PPT), maximal isokinetic muscle strength (MIMS), and active range of motion (ROM) of females with persistent pain ≥1.5 years after breast cancer treatment (BCS) compared with pain-free matched controls (CON), and examine the presence of movement-evoked pain (MEP) during assessment of MIMS.

**Methods:**

The PPTs of 18 locations were assessed using a pressure algometer and a numeric rating scale was used to assess intensity of MEP. Active ROM and MIMS were measured using a universal goniometer and an isokinetic dynamometer, respectively.

**Results:**

A two-way analysis of variance revealed that PPTs across all locations, MIMS for horizontal shoulder extension/flexion and shoulder adduction, active ROM for shoulder flexion, horizontal shoulder extension, shoulder abduction, and external shoulder rotation were significantly lower for BCS compared with CON (*P* < 0.05). MEP was significantly higher for BCS and MEP intensity had a significant, negative correlation with PPTs (*P* < 0.01).

**Discussion/conclusion:**

BCS with persistent pain ≥1.5 years after treatment demonstrates widespread reductions in PPTs and movement-specific reductions in MIMS and active ROM of the affected shoulder, along with MEP during physical performance assessment.

**Implications for cancer survivors:**

BCS with persistent pain ≥1.5 years after treatment shows signs of central sensitization and may benefit from individualized rehabilitation.

**Supplementary Information:**

The online version contains supplementary material available at 10.1007/s11764-021-00995-y.

## Introduction

Persistent pain after treatment for breast cancer is a common problem affecting 25–60% of the patients for years after the initial treatment [1]. Persistent pain refers to pain in and around the area of surgery (e.g., chest, shoulder, arm, and side of body) lasting more than 3 months [2] that often cause considerable physical disability [3]. Pain after treatment for breast cancer is a frequent source of decreased physical function [4], and is a primary cause of upper limb impairments [4], which lead to limitations in activities of daily living [5] as well as reduced quality of life. Furthermore, pain has been associated with mechanical hyperalgesia [6] and loss of strength and range of motion (ROM) in the affected shoulder [7], which may further exacerbate upper limb impairments [8]. Upper body strength and ROM contribute to the functional status by promoting adequate scapula-humeral mobility and stability [9], and hence, it could be speculated that pain alters functional shoulder recovery in patients. Indeed, pain intensity and sensitivity have been associated with upper limb dysfunction one and a half years after treatment for breast cancer [10], suggesting that persistent pain has long lasting negative effects on shoulder function in breast cancer survivors (BCS). However, De Groef et al. [10] are the only study to elaborate on the long-term contribution of pain to shoulder dysfunction in BCS, that we are aware of, and little is known about the influence of pain on shoulder function beyond 1.5 years after the initial treatment.

Importantly, kinesiophobia was recently reported as the main contributor to-pain related disability in mid- to long-term BCS [11], implying that BCS experience persistent pain during the performance of physical tasks, i.e., movement-evoked pain (MEP). Movement-evoked pain refers to pain that is experienced in response to a physical challenge [12] and can influence force production through activation of the nociceptors and of the pain processing network in the brain [13]. Consequently, MEP can inhibit muscular strength and impair movement. Furthermore, higher MEP have previously been associated with lower levels of physical performance and increased mechanical pain sensitivity in patients with painful knee osteoarthritis [14]. Therefore, assessment and consideration of MEP could potentially increase the clinical value of shoulder function tests and pain assessments in BCS. However, although abundant evidence exists for the impact of pain on physical functional performance [14], no studies to date have examined MEP during functional physical performance assessments, such as shoulder strength, among BCS with persistent pain after treatment. Moreover, to the best of our knowledge, no studies have assessed pain perception and shoulder function simultaneously in BCS with persistent pain beyond 1,5 years after treatment, despite evidence of shoulder dysfunction and pain after treatment for breast cancer persisting for up to 6 [15] and 8 [1] years respectively.

Therefore, the purpose of this study was 3-fold; (1) to assess pressure pain sensitivity, shoulder strength, and ROM in BCS with self-reported pain ≥1.5 years after treatment compared with asymptomatic female controls; (2) investigate the presence of MEP during the assessments of shoulder strength; and (3) determine if this is related to mechanical pain sensitivity. We hypothesized that BCS compared with matched controls would be characterized by deficits in shoulder function, i.e., mechanical hyperalgesia, lower levels of shoulder strength, and active ROM, and exhibit MEP related to mechanical hyperalgesia. Such information is a pre-requisite for proper design and development of specific exercise interventions and rehabilitation programs for efficient rehabilitation and recovery of shoulder function and pain management in BCS.

## Methods

### Participants

Forty-two women participated in this case-control study, equally distributed in two groups: BCS (*N* = 21, mean (CI: 95%); age (years) = 57.4 (54;60.8), height (cm) = 167.9 (165.5;170.2), body mass index (kg/m^2^) = 27.6 (25.3;29.8)), and CON (N = 21, mean (CI: 95%); age (years) = 60 (55.5;64.5), height (cm) = 165.5 (162.8;168.2), body mass index (kg/m^2^) = 27.1 (24.9;29.2)). The same BCS group participated in a separate reliability study assessing absolute and relative reliability of pressure pain threshold (PPT), maximal isokinetic muscle strength (MIMS), and active ROM [16]. Participants for BCS were recruited by means of a database letter through the national database managed by the Danish Breast Cancer corporate Group. Women were eligible for inclusion in the BCS group if they were as follows: (i) diagnosed with primary unilateral breast cancer (grades I–IIIA); (ii) adult women at least 18 years of age; (iii) treated for breast cancer (i.e., surgery and possible adjuvant chemo and/or radiotherapy) at least 18 months before the start of the study; (iv) experiencing pain (self-reported) in the areas of the breast, shoulder, axilla, arm, and/or side of body with an intensity of ≥3 on a numeric rating scale (0 = no pain, 10 = worst pain imaginable); (v) women without signs of cancer recurrence; and (vi) reading, writing, and speaking Danish. Reasons for ineligibility were as follows: (i) breast surgery for cosmetic reasons or prophylactic mastectomy; (ii) bilateral breast cancer; (iii) recurrence of cancer; (iv) lymphedema; (v) other adverse medical conditions with potential influence on the study, or (vi) previous diagnosis of fibromyalgia syndrome. Controls were asymptomatic (pain-free) female volunteers with no previous history of cancer matched to the BCS group as well possible for age (mean difference, range: 2.6, 0–7 years) and body mass index (mean difference, range: 0.5, 0.0–2.2 kg/m^2^). Women were eligible for inclusion in the control group if they were as follows: (i) adult women at least 18 years of age and (ii) reading, writing, and speaking Danish. Reasons for ineligibility were as follows: (i) pregnancy; (ii) drug addiction, e.g., continued use of cannabis, opioids, or other substances taken for a non-medical purpose; (iii) presence of signs or symptoms of musculoskeletal pain; (iv) history of persistent pain or trauma in the upper body; (v) adverse medical conditions with potential influence on the study (e.g., chronic fatigue syndrome); (or vi) participation in other pain trials throughout the study period. All the participants were instructed to avoid physical activity and consumption of alcohol, caffeine, nicotine, or analgesics (e.g., paracetamol, ibuprofen, or codeine) in the last 24 h prior to the experiments. The study was conducted in accordance with STROBE guidelines [17]. The study protocol was approved by the local Ethics Committee (N-20180090) and conducted according to the Declaration of Helsinki. Following a detailed written and verbal explanation of the experimental risks, the participants gave their written informed consent prior to participating in the study.

### Study design

**Fig. 1:**
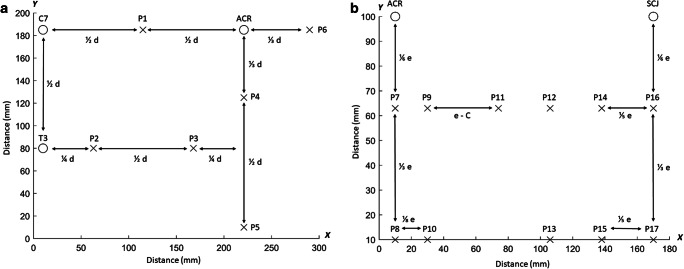
Pressure pain threshold grids. Schematic representation of the dorsal (**a**) and ventral (**b**) grids for pressure pain threshold (PPT) assessments. The PPTs of the dorsal region were measured over 6 points located on the trapezius muscle (P1-P2), infraspinatus (P3), posterior deltoid (P4), latissimus dorsi (P5), and lateral deltoid (P6). The PPTs of the ventral region were measured over 11 points located on the anterior deltoid (P7-P10) and pectoralis major (P11-P17). d = distance between the seventh cervical vertebra (C7) and acromion (ACR), e = distance between the sternoclavicular joint (SCJ) and acromion (ACR), and C = the summed distance between P11 and P12, P12 and P14, and P14, and P16 on the x-axis

Each participant completed a familiarization session (FS), and an experimental session (ES) separated by 1 week (Fig. 1). Baseline characteristics (anthropometrics, demographics, physical, health, surgical, medical, and pain profile) and physical activity level (IPAQ) [18] were collected during FS only, while PPT, active ROM, and MIMS were measured during both FS and ES (Fig. 1). PPT, active ROM, and MIMS were collected during FS for familiarization purposes only and not included in the further analysis. To avoid a confounding influence of excessive fatigue during the experimental sessions, measurements were performed unilaterally on the operated side for BCS and the left/right distribution were matched for CON. The resulting no. of assessments performed on the dominant side were 9 (34%) and 8 (38%) for BCS and CON, respectively. The participants were blinded to the measures of PPT, active ROM, and MIMS, and all assessments were performed by the same researcher who had received weekly training in the three months leading up to the study (GHFR, not blinded).

### Outcomes

Movement evoked pain (MEP) intensity was rated after each series of MIMS on a 0–10 Numeric Pain Rating Scale (NPRS), where 0 corresponded to “no pain” and 10 to “worst pain imaginable” [19]. The cut-off scores used as reference were as follows: 0 = no pain; 1–3 = mild; 4–6 = moderate; 7–10 = severe pain [20].

Pressure pain thresholds are a reliable measure of mechanical pain sensitivity in BCS with intra class correlation coefficients (ICC) ranging from 0.88 to 0.97 [16], and were obtained in agreement with Rasmussen et al. [16]. Hence, PPTs were measured unilaterally across 17 points, located on the dorsal (6 points) and ventral (11 points) parts of the chest, shoulder, and neck region (Fig. 1). In addition, a distant reference point was located on the ipsilateral tibialis anterior muscle to assess the potential presence of generalized hyperalgesia [21]. All PPT measurements were collected using a pressure algometer (Somedic AB, Farsta, Sweden) with a 1 cm^2^ probe and a constant incremental pressure rate of 30 kPa/s. The participants were instructed to press a handheld button immediately when the sensation changed from pressure to pain. The measurements were performed twice in predefined order (P1-18), and a third time if the coefficient of variance was ≥20%. There were approx. 6 min between measurements made over the same point to avoid temporal summation of pain. Pressure pain threshold map of the dorsal and ventral regions was constructed from the mean PPT values of the points located on the dorsal and ventral muscles respectively by measuring the distance *d* between C7 and acromion, and the distance *e* between acromion and the sternoclavicular joint for each participant and computing the inter-distance between points. Inverse distance–weighted interpolation was then applied to obtain a map of the spatial pressure pain distribution of each region [22].

Body mass index (kg/m^2^) was calculated from height and body mass measured at the familiarization session. The fat-free mass (FFM), body fat mass (BFM), skeletal muscle mass (SMM), and body fat percentage (BF%) of each participant were computed using direct segmental multifrequency (DSM) bioelectrical impedance analysis (BIA) (InBody 370, Biospace, Seoul, Korea), in agreement with the manufacturer’s recommendations. DSM-BIA is considered valid and reliable for body composition measures in the general population when compared to dual-energy x-ray absorptiometry (DXA) [23].

Active ROM was measured with a universal goniometer for six movement directions: (1) supine shoulder flexion, (2) supine horizontal shoulder flexion, (3) horizontal shoulder extension, (4) seated upright shoulder abduction, (5) supine internal shoulder rotation, and (6) supine external shoulder rotation in agreement with the protocol of Rasmussen et al. [16]. Supine measurements were performed with the knees flexed to flatten the lumbar spine, and with manual stabilization of the scapulae during assessment of inter/external shoulder rotation. For the seated upright measurements, participants were positioned seated firmly against the back of the chair to ensure trunk stabilization, and instructed to maintain a neutral head position. For further details on anatomical starting positions of the shoulder and arm, see Rasmussen et al. [16]. Goniometric measurements of active ROM are reliable in BCS (ICCs: 0.66–0.97) [16], and were performed by the same researcher by aligning the goniometer arms between bony landmarks (e.g., olecranon process and ulnar styloid of the forearm, and medial epicondyle of the humerus) and positioning the fulcrum of the goniometer above the approximate projection of the given joint center on the movement plane. Similar to PPT, the assessments were performed twice over two rounds in systematic order and a third time if the measurements had a coefficient of variance ≥20%. The mean ROM values were calculated for each movement direction.

Maximal isokinetic muscle strength (MIMS) can also be measured reliably in BCS (ICCs: 0.62–0.92) [16], and was collected for eight movement directions: (1) supine shoulder flexion, (2) supine shoulder extension, (3) supine horizontal shoulder extension, (4) supine horizontal shoulder flexion, (5) seated shoulder abduction, (6) seated shoulder adduction, (7) supine internal shoulder rotation, and (8) supine external shoulder rotation. All measurements were performed using an isokinetic dynamometer (Humac Norm, model 770, Computer Sports Medicine Inc., Stoughton, USA). Each participant was familiarized with the protocol during FS, and performed a brief (approx. 10 min) general warm up of various stretching exercises for the prime movers prior to testing. This was followed by a series of 10 consecutive contractions with submaximal progressive effort followed by a series of five consecutive contractions at maximal effort for each muscle group with a 2-min rest period between series. Rest between measurements for each movement direction consisted of the time required for readjustment of the dynamometer (approximately 5 min). All isokinetic strength testing was conducted at a speed of 60^0^/s through the ROM previously measured for each movement direction. The first repetition of each maximal trial was discarded, and mean peak torque was computed for the remaining four at a fixed joint angle. Mean peak torque was then normalized to FFM and expressed as Nm/kg FFM. Gravity correction was performed for each participant prior to the series of assessments. For a more detailed description about strength measurements, see Rasmussen et al. [16].

### Statistical analysis

The minimum required sample size to detect a significant difference in PPT between groups, assuming an alpha level of 0.05, a beta level of 0.80 and a large effect size of 0.52 estimated from the pectoral PPTs reported by Caro-Moran et al. [6], was determined to be at least 32 (16 per group). To account for a potential drop out of 20–30%, 42 participants were enrolled in the study. Independent samples *t* tests were used to compare age and BMI between groups. A two-way analysis of variance (ANOVA) was performed to investigate potential differences between groups. PPT was used as dependent factor with anatomical location (P1-18, mean dorsal, & mean ventral) and group (BCS, CON) as independent factors. Similarly, active ROM and MIMS were used as dependent factors with movement direction (1–6 & 1–8) and group (BCS, CON) as independent factors. In the case of a significant interaction effect, simple main effects were analyzed within each combination of the other effects through univariate tests based on linearly independent pairwise comparisons among the estimated marginal using the overall error term of the two-way ANOVA. If no significant interaction effect was found, main effects were reported. Post hoc analyses were performed as univariate analyses with Bonferroni correction for multiple comparisons = *α*/*n*. The Shapiro-Wilks test of normality was applied to test the assumption of normal distribution and homogeneity of variance was tested through Levene’s Test of Equality of Error Variances. If the assumptions of normality and equal variance were violated, a Mann-Whitney *U* rank based nonparametric test with Holm-Bonferroni sequential correction for multiple comparisons was applied to assess between group differences for each outcome measure = *α* / (*n* – rank +1). Associations between PPTs and MEP assessments were explored through a Pearson's product-moment correlation analysis or Spearman’s rank order correlation. Assumptions of linearity and outliers were determined from visual inspection and the Shapiro-Wilks test of normality was applied to test the assumption of normal distribution. Since the afore-mentioned assumptions were violated, only Spearman’s rank order correlation was applied. All statistical procedures were conducted in IBM SPPS Statistics (26.0 version; IBM Corp., Armonk, NY, USA). A *P* value less than 0.05 was considered statistically significant. Differences are expressed as mean (confidence interval (CI) 95%) and median (25–75^th^ percentile) for parametric and non-parametric tests, respectively. Effect size estimates are reported as partial eta squared (partial *η*^2^), and interpreted according to Cohen [24] in which ≥0.01 to < 0.06, ≥ 0.06 to < 0.14 and ≥ 0.14 correspond to small, medium, and large effect sizes, respectively. Similarly, correlation coefficients were interpreted in accordance with the guidelines of Cohen, where >0.1 to < 0.3, 0.3 to <0.5 and > 0.5 denote a small, moderate, or large correlational effects, respectively. Missing data points were omitted from the above analyses.

## Results

The independent sample *t* tests revealed no statistically significant difference between groups for age (*t* (− 0.974), *P* = 0.336, partial *η*^2^ = 0.01) and BMI (*t* (0.338), *P* = 0.737, partial *η*^2^ = 0.00). The sociodemographic, physical, and health characteristics of BCS and CON are reported in the supplementary information (Appendix). For detailed information on surgical, medical, and pain profile of BCS, see supporting material for Rasmussen et al. [16].

**Fig. 2:**
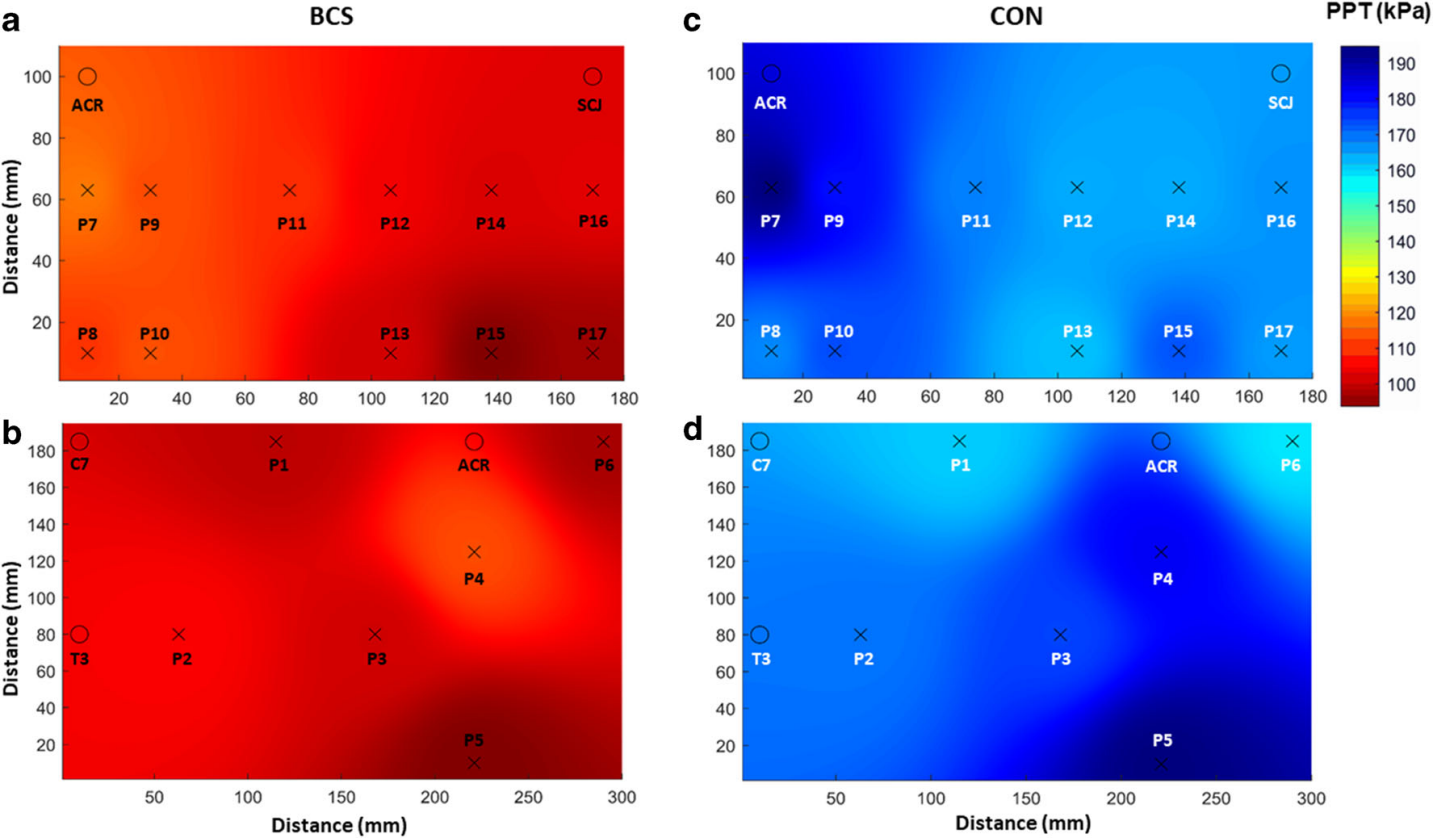
Pressure pain threshold maps. Mean pressure pain threshold (PPT) maps from the eleven anatomical locations on the ventral shoulder region, and six locations on the dorsal shoulder region of the BCS (**a** and **b**) and control (**c** and **d**) groups. Note the significantly lower PPT for BCS compared with CON (*p* < 0.01)

Mean PPTs for BCS and CON are reported in Table 1. The two-way ANOVA revealed no significant interaction effect between group and location, (*F* (19,800) = 0.683, *P* = 0.838, partial *η*^2^ = 0.016)*.* A significant main effect was found for group (*F* (1800) = 375,951, *P* = ≤ 0.001, partial *η*^2^ = 0.320) and location (*F* (19,800) = 6062, *P* ≤ 0.001, partial *η*^2^ = 0.126). The PPTs were on average 49% lower for BCS than for CON across all locations (mean (95% CI) = − 136.3 kPa (− 150.1: − 122.5)). This group difference was further visualized by the PPT maps (Fig. 2), which also revealed similar spatial distribution of mechanical pain sensitivity between groups. The most sensitive area was located on the inferior portion of the pectoralis major on the ventral region for both groups, whereas the least sensitive areas were located at the posterior deltoid and the latissimus dorsi on the dorsal region for BCS and CON respectively (Fig. 2).

**Table 1:** Mean pressure pain threshold location across anatomical locations

PPT locations (kPa)	BCS (*N* = 21) Mean (95% CI)	CON (*N* = 21) Mean (95% CI)	Group difference Mean (95% CI)	Effect size (partial *η*^2^)
Point 1	164.2 (130.0; 198.3)	300.0 (241.3; 358.7)	− 135.8 (− 201.6; − 70.1)*	0.023
Point 2	178.1 (135.6; 220.6)	319.2 (257.8; 380.5)	− 141.1 (− 213.4; − 68.8)*	0.025
Point 3	168.6 (135.4; 201.8)	329.9 (259.5; 400.2)	− 161.2 (− 236.6; − 85.8)*	0.032
Point 4	194.7 (144.7; 244.6)	346.2 (268.0; 424.3)	− 151.5 (− 241.4; − 61.6)*	0.028
Point 5	151.3 (117.6; 185.1)	372.2 (295.7; 448.6)	− 220.8 (− 301.7; − 139.9)*	0.058
Point 6	159.3 (119.9; 198.7)	294.2 (241.5; 346.8)	− 134.9 (− 198.6; − 71.1)*	0.022
Mean.Dors	169.4 (130.4; 208.4)	326.9 (260.2; 393.6)	− 157.6 (− 219.3; − 95.8)*	0.030
Point 7	139.4 (115.4; 163.5)	284.2 (222.6; 345.9)	− 144.8 (− 208.9; − 80.7)*	0.026
Point 8	126.7 (103.9; 149.4)	234.4 (190.7; 278.0)	− 107.7 (− 155.4; − 60.0)*	0.014
Point 9	131.1 (106.5; 155.7)	257.3 (205.4; 309.2)	− 126.2 (− 181.8; − 70.5)*	0.020
Point 10	131.9 (104.1; 159.7)	245.7 (204.7; 286.8)	− 113.9 (− 161.9; − 65.8)*	0.016
Point 11	125.6 (92.1; 159.0)	235.9 (188.5; 283.4)	− 110.4 (− 166.6; − 54.1)*	0.015
Point 12	114.5 (78.2; 150.9)	227.9 (185.0; 270.8)	− 113.4 (− 167.9; − 58.9)*	0.016
Point 13	109.0 (70.6; 147.3)	223.9 (181.1; 266.8)	− 115.0 (− 170.7; − 59.3)*	0.016
Point 14	110.6 (88.6; 132.7)	227.9 (180.3; 275.5)	− 117.3 (− 168.1; − 66.5)*	0.017
Point 15	93.6 (73.8; 113.3)	244.4 (192.9; 295.9)	− 150.8 (− 204.2; − 97.3)*	0.028
Point 16	112.2 (85.0; 139.4)	232.5 (184.4; 280.7)	− 120.3 (− 173.9; − 66.7)*	0.018
Point 17	99.4 (74.5; 124.3)	230.9 (176.0; 285.7)	− 131.5 (− 189.9; − 73.1)*	0.021
Mean.Vent	117.6 (89.5; 145.7)	240.5 (192.1; 288.9)	− 122.8 (− 184.6; − 61.1)*	0.027
Reference point	193.9 (154.1; 233.7)	343.2 (267.9; 418.5)	− 149.3 (− 211.0; − 87,6)*	0.019

*PPT* pressure pain threshold, *Mean.Dors* mean dorsal shoulder, *Mean.Vent* mean ventral shoulder, *CI* confidence interval, *BCS* breast cancer survivors, *CON* controls

*P < 0.001 (post hoc analyzes: differences in PPT between groups)

Mean active ROM for BCS and CON are reported in Table 2. The two-way ANOVA revealed a significant interaction effect between group and movement, (*F* (5240) = 3515, *P* = 0,004, partial *η*^2^ = 0.068), demonstrating that group differences in active ROM were movement dependent and reduced for BCS in some, but not all movement directions (see Table 3)*.* Simple main effects analyses showed that mean active ROM of BCS was significantly lower when compared to CON for flexion (*F* (5240) = 10,066, *P* = 0.002, partial *η*^2^ = 0.040), horizontal extension (*F* (5240) = 16,793, *P* ≤ 0.001, partial *η*^2^ = 0.065), abduction (*F* (5240) = 5935, *P* = 0.016, partial *η*^2^ = 0.024), and external rotation (*F* (5240) = 20,261, *P* ≤ 0.001, partial *η*^2^ = 0.078). Active ROM was on average 10% lower for BCS than for con across these movement directions.

**Table 2:** Mean active range of motion across movement directions

Active shoulder ROM (^0^)	BCS (*N* = 21) Mean (95% CI)	CON (*N* = 21) Mean (95% CI)	Group difference Mean (95% CI)	Effect size (partial *η*^2^)
Flexion	158.5 (151.3; 165.7)	171.2 (167.8; 174.6)	− 12.7 (− 20.4; − 5.0)**	0.040
Hor. flexion	21.5 (19.5; 23.4)	21.1 (19.3; 23.0)	0.3 (− 2.3; 2.9)	0.000
Hor. extension	97.5 (90.1; 104.8)	113.9 (110.7; 117.1)	− 16.5 (− 24.2; − 8.7)*	0.065
Abduction	144.2 (134.8; 153.6)	154 (145.4; 162.6)	− 9.8 (− 22.1; 2.5)***	0.024
Internal rotation	43.2 (37.2; 49.3)	45.3 (42.5; 48.1)	− 2.1 (− 8.5; 4.4)	0.001
External rotation	72.4 (65.8; 79.1)	90.5 (84.5; 96.5)	− 18.1 (− 27.8; − 9.4)*	0.078

*ROM* range of motion, *CI* confidence interval, *BCS* breast cancer survivors, *CON* controls

^*0*^Joint angle

*P < 0.001; **P < 0.01; ***P < 0.05 (post hoc analyses: differences in ROM between groups)

**Table 3:** Mean maximal isokinetic muscle strength across movement directions

Fixed position normalized peak torque (Nm/kg FFM)	BCS (*N* = 20) Mean (95% CI)	CON (*N* = 21) Mean (95% CI)	Group difference Mean (95% CI)	Effect size (partial *η*^2^)
Extension (63^0^)	0.47 (0.44; 0.50)	0.49 (0.45; 0.54)	− 0.03 (− 0.08; 0.03)	0.002
Flexion (63^0^)	0.53 (0.48; 0.58)	0.54 (0.49; 0.58)	− 0.01 (− 0.07; 0.05)	0.000
Hor. extension (33^0^)	0.43 (0.37; 0.49)	0.54 (0.50; 0.59)	− 0.12 (− 0.19; − 0.05) *	0.044
Hor. flexion (8^0^)	0.56 (0.48; 0.63)	0.66 (0.61; 0.70)	− 0.10 (− 0.18; − 0.01)**	0.032
Abduction (58^0^)	0.55 (0.49; 0.61)	0.51 (0.47; 0.56)	0.04 (− 0.03; 0.11)	0.004
Adduction (58^0^)	0.40 (0.36; 0.45)	0.55 (0.51; 0.59)	− 0.15 (− 0.21; − 0.09) *	0.069
Internal rotation (11^0^)	0.25 (0.22; 0.29)	0.29 (0.27; 0.30)	− 0.03 (− 0.07; 0.00)	0.003
External rotation (23^0^)	0.25 (0.21; 0.28)	0.26 (0.24; 0.29)	− 0.02 (− 0.06; 0.03)	0.001

^0^Joint angle from anatomical zero, *Nm* Newton meter, *FFM* fat-free mass, *CI* confidence interval, *BCS* breast cancer survivors, *CON* controls

*P < 0.001; **P < 0.01(post hoc analyzes differences in MIMS between groups)

Mean MIMS for BCS and CON is reported in Table 3. The two-way ANOVA revealed a significant interaction effect between group and movement, (*F* (7312) = 4116, *P* ≤ 0.001, partial *η*^2^ = 0.085) demonstrating that group differences in MIMS were movement dependent and reduced for BCS in some, but not all movement directions (see Table 3). Simple main effect analyses showed that mean MIMS of BCS was significantly lower when compared to CON for horizontal shoulder extension (*F* (1312) = 14,314, *P* ≤ 0.001, partial *η*^2^ = 0.044), horizontal shoulder flexion (*F* (1312) = 10,303, *P* = 0.001, partial *η*^2^ = 0.032), and shoulder adduction (*F* (1312) = 23,232, *P* ≤ 0.001, partial *η*^2^ = 0.069). MIMS was on average 11% lower for BCS than for con across these movement directions.

The Mann-Whitney *U* test revealed that intensity of MEP was significantly higher for BCS when compared to CON during shoulder extension/flexion (adj. *P* = 0.004, partial *η*^2^ = 0.269), horizontal shoulder extension/flexion (adj. *P* = 0.004, partial *η*^2^ = 0.276), shoulder abduction/adduction (adj. *P* = 0.004, partial *η*^2^ = 0.272), and for internal/external shoulder rotation (adj. *P* = 0.004, partial *η*^2^ = 0.301). Group difference in median [25th; 75th percentiles] was 4.0 [0.0; 5.8] during shoulder extension/flexion, 4.0 [0.0; 7.0] during horizontal shoulder extension/flexion, 4.5 [0.0; 5.0] during abduction/adduction, and 4.5 [0.3; 5.8] during internal/external rotation. The Spearman’s rank order correlation revealed a significant, large negative correlation effect between the intensity of movement evoked pain and the grand mean PPT in BCS for horizontal flexion/extension MEP (*ρ* [18], − 0.572, *P* = 0.008), and abduction/adduction MEP (*ρ* [18], − 0.536, *P* = 0.015), but not for flexion/extension MEP (*ρ* [18], − 0.244, *P* = 0.299) and internal/external rotation MEP (*ρ* [18], − 0.284, *P* = 0.225).

## Discussion

This study examined differences between BCS with persistent pain ≥1.5 years after treatment and asymptomatic controls for mechanical pain sensitivity, shoulder strength, active ROM, and MEP. As hypothesized, BCS with self-reported pain ≥1.5 years after treatment demonstrated significant reductions in pressure pain thresholds (− 49%), maximal isokinetic muscle strength (− 11%), and active ROM (− 10%) in the affected shoulder. The effect size (partial *η*^2^) for interaction- and main effects was 0.320 (large) for PPT, 0.068 (moderate) for active ROM, and 0.085 (moderate) for MIMS. Furthermore, the shoulder strength assessments elicited a moderate movement evoked pain response in BCS (i.e., NPRS = 4–6), which had a large negative correlation effect with pressure pain thresholds. These results provide further evidence that pain and loss of function in the affected shoulder are long-lasting adverse effects to breast cancer treatment and indicate that BCS ≥1.5 years post-treatment may benefit from specific exercise training for the shoulder girdle. Furthermore, the results of this study provide preliminary evidence that assessment of physical performance can elicit an MEP response in BCS with persistent pain after treatment. This response appears to be associated with the extent of mechanical pain sensitivity and may represent a relevant clinical outcome to monitor when investigating physical function and prescribing physical activity and exercise for this population.

### Pain

The PPTs observed in the present study are similar to those previously reported for BCS [6] and healthy individuals [25]. In agreement with Caro-Moran et al. [6], we found that the PPTs of BCS were considerably lower across all anatomical locations including a remote point over the tibialis anterior muscle when compared with CON, which demonstrate a widespread mechanical hyperalgesia. These differences in PPT were greater than the minimum detectable change (MDC) previously reported to range from 33.2 to 78.2 kPa [16], demonstrating a clinically significant increase in mechanical pain sensitivity in BCS. This has previously been suggested as indicative of a central sensitization mechanism in BCS with pain after treatment [6], a notion which is further supported by the marked reduction in PPT measured distant to the surgical area (i.e., tibialis anterior). Hence, the similar spatial mechanical pain sensitivity between groups, as illustrated by the PPT maps (Fig 2), could be speculated to reflect an otherwise normal difference in spatial PPT distribution between shoulder regions for BCS, which is amplified by alterations in pain- modulatory processes [26]. In contrast, potential nerve damage from the surgical and/or adjuvant therapy has previously been suggested as possible explanations [21], implying a more localized source of the greater sensitivity observed for the ventral shoulder region in BCS.

Sign of central sensitization could also explain the MEP response reported for BCS for all movement directions when measuring muscle strength, as centrally-mediated pain pathways are implicated in MEP [27]. Consequently, discomfort associated with strenuous physical activity could intensify to perceived pain, or an exacerbation of perceived pain. This notion is supported by the strong, significant correlations observed between PPT and MEP for horizontal extension/flexion and abduction/adduction, indicating that greater mechanical pain sensitivity is associated with higher intensity of MEP in BCS. Thus, the mechanical pain sensitivity (PPT) and MEP observed in the present study could be interpreted as further evidence for the presence of central sensitization in women with persistent pain after breast cancer treatment. Therefore, MEP during physical performance assessments of patients with signs of central sensitization, such as BCS with persistent pain, may be a relevant clinical outcome to measure in order to improve our understanding of the influence of central sensitization pain on physical function and task performance.

In addition, monitoring MEP could be useful to determine the appropriate dose of exercise in this population and possibly reduce or avoid fear of movement that may otherwise result in pain related disability in mid- to long-term BCS [11]. As highlighted by Campbell et al. [28], there is currently little evidence to guide the appropriate modality (strength, aerobic, flexibility, etc.) or dose (volume, intensity, frequency, etc.) of exercise for cancer survivors, and both too little and too much exercise could be suboptimal for tissue health and/or pain management [27]. This point may be particularly important when prescribing exercise or physical/leisure activities for pain conditions with movement-associated fear-avoidance behavior as inappropriate exercise dosage may cause symptom exacerbation [29]. Therefore, monitoring the magnitude of MEP elicited by the chosen modality and dosage of exercise may provide valuable information on the efficacy of a training intervention aiming at reducing pain and improving shoulder function in BCS with persistent pain as general recommendations may not be appropriate. Furthermore, this approach could be useful to guide the rehabilitation early in the cancer treatment continuum, where exercise may limit treatment-related upper extremity impairments [30].

### Shoulder strength and active range of motion

The results of this study demonstrate a significant reduction in strength and active ROM of the affected shoulder in BCS ≥1.5 years beyond treatment as compared with healthy controls. Our findings also provide preliminary evidence that strength and ROM remain affected in some movement directions like horizontal shoulder extension, but may have recovered to normal levels in others like internal shoulder rotation. The observed reductions were greater than the previously reported MDCs for active ROM (MDC: 8.4 −20.8°) in shoulder flexion and horizontal shoulder extension, and for MIMS (MDC 0.09–0.19 Nm/kg FFM) in shoulder adduction [16], and are considered clinically significant for these movement directions. These movement-specific impairments are different from previous studies who report a general reduction in shoulder strength and ROM in BCS regardless of movement direction [31–36], which may be largely explained by differences in methodological approaches and design between studies. For example, only a few other case-control studies have evaluated shoulder function after breast cancer treatment in comparison to healthy controls [35, 36]. In contrast, several studies have employed a cross-sectional design for assessing shoulder strength and ROM (both active and passive) in BCS by comparing the affected and unaffected limbs [31, 32, 34]. However, this approach may underestimate the loss of shoulder function as shoulder morbidity after breast cancer can be bilateral [15] and makes it difficult to distinguish longer-term treatment-related reductions from age-related decline.

Another potential explanation for the observed differences in shoulder impairment from previous investigations may be variability in proximity to the treatment when obtaining the measurements. For example, this study was performed on average 66.1 months post-treatment compared with approximately 6 months [35], 18 months [10], 32 months [31], 44 months [32], 47 months [33] and 51 months [36] in previous investigations. Greater shoulder impairment has been reported closer to the treatment [7]. Hence, it could be speculated that the movement-specific impairments observed in this study are a product of variable long-term recovery for the tissues involved. Muscles such as the pectorals are strongly affected by surgery and/radiotherapy for breast cancer, which can cause scar tissue formation and soft tissue fibrosis [37]. This can impair normal gliding between skin-related structures, fasciae, and muscles [9] and may never completely disappear [37]. In contrast, muscles affected indirectly such as the rhomboid and trapezius muscles may have recovered to normal levels at 66.1 months after breast cancer treatment, despite significant impairments in closer proximity to treatment [38]. However, all of the affected movement directions in this study involved the pectorals, and thus, reduced pectoralis minor- and major muscle flexibility and contractility from permanent scar tissue formation could be a potential cause for the movement-specific loss of an active ROM.

### Strengths and limitations

The current study is the first to our knowledge to examine pain and shoulder function concurrently in BCS with persistent pain ≥1.5 years after treatment. In addition, we demonstrate for the first time that this population exhibit MEP in the affected shoulder and that intensity of MEP has a strong negative correlation with mechanical pain sensitivity. However, this study is not without limitations. The measurements were only performed unilaterally on the operated side for BCS and future studies should also investigate if the widespread mechanical hyperalgesia and the decreases in shoulder function are bilateral as indicated in previous studies [6, 15]. For example, the inclusion of self-reported multidimensional pain scales, such as the central sensitization index or the pain sensitivity questionnaire could have complimented our findings but none of these measures have been validated in Danish. Further, pain can fluctuate over time, [39] and consequently, the results of the present study are only representative of the pain experienced by each participant at the time of assessment. Finally, multiple non-treatment risk factors for persistent pain in BCS have been identified [40]; therefore, due to the cross sectional study design, the results of this study cannot be used to infer causality between treatment for breast cancer and pain.

### Conclusion

The results of the present study showed that BCS with persistent pain ≥1.5 years after treatment demonstrate clinically significant reductions in pressure pain thresholds, strength, and active ROM and exhibit greater MEP when compared with asymptomatic controls. The widespread mechanical hyperalgesia can be interpreted as sign of central sensitization in BCS with persistent pain, and was negatively correlated with the intensity of MEP during assessment of shoulder strength. Hence, assessing MEP may be of relevance to clinicians when investigating physical function and performance in BCS with persistent pain after treatment. Further, the movement-specific deficits observed for shoulder strength and active ROM in this population imply that the muscles involved in these movement directions may benefit from being targeted directly through strengthening exercise, such as resistance training.

### Supplementary information

Appendix. Cohort description

ESM 1 (DOCX 18 kb)

## Declarations

### Disclaimer

The funders had no role in study design, data collection, and analysis, decision to publish, or preparation of the manuscript. The results of the study are presented clearly, honestly, and without fabrication, falsification, or inappropriate data manipulation.
