# Supplementary material for: Pain sensitivity and shoulder function among breast cancer survivors compared to matched controls: a case-control study
Source: J Cancer Surviv. 2021 Jan 26;17(1):150–9. doi: 10.1007/s11764-021-00995-y (PMC9970942; doi:10.1007/s11764-021-00995-y)
Supplement: Supplementary file 1 — (DOCX 18 kb) [file 11764_2021_995_MOESM1_ESM.docx]

**Table 1:** **Characteristics of the 42 women participating in this study.**

| **SOCIODEMOGRAPHIC AND PHYSICAL PROFILE**: | **BCS (N = 21)** | **CON (N = 21)** |
| --- | --- | --- |
| **Age, mean (CI: 95%), y** | 57.4 (54;60.8) | 60 (55.5;64.5) |
| **Height, mean (CI: 95%), cm** | 167.9 (165.5;170.2) | 165.5 (162.8;168.2) |
| **Living arrangement, No. (%)** |  |  |
| Living with a partner | 17 (81) | 15 (71) |
| Living alone | 3 (14) | 6 (21) |
| Other | 1 (15) | 0 (0) |
| **Education, No. (%)** |  |  |
| Short | 6 (26) | 4 (19) |
| Medium | 14 (67) | 13 (62) |
| Long | 1 (5) | 4 (19 |
| Other | 0 (0) | 0 (0) |
| **Employment, No. (%)** |  |  |
| Full time | 11 (52) | 10 (48) |
| Part time | 3 (14) | 2 (10) |
| Staying at home | 0 (0) | 0 (0) |
| Retired | 6 (29) | 8 (38) |
| Sick leave | 0 (0) | 0 (0) |
| Other | 1 (5) | 1 (5) |
| **Body mass index, mean (CI: 95%), kg/m^2^** | 27.6 (25.3;29.8) | 27.1 (24.9;29.2) |
| **Body mass index, No. (%)** |  |  |
| ≤ 25 kg/m^2^ | 8 (38) | 7 (33) |
| >25 - ≤30 kg/m^2^ | 6 (29) | 10 (48) |
| >30 kg/m^2^ | 7 (33) | 4 (19) |
| **Menopausal status, No. (%)** |  |  |
| Pre | 1 (5) | 4 (19) |
| Peri | 4 (19) | 4 (19) |
| Post | 16 (76) | 13 (63) |
| **HEALTH BEAVIOUR:** |  |  |
| **Level of physical activity, No. (%)** |  |  |
| Low | 6 (29) | 4 (19) |
| Moderate | 10 (48) | 15 (71) |
| High | 5 (24) | 2 (10) |
| **Smoking, No. (%)** |  |  |
| Current smoker | 1 (5) | 0 (0) |
| Exsmoker | 11 (52) | 7 (33) |
| Never smoker | 9 (43) | 14 (67) |
| **Alcohol consumption** |  |  |
| No. units per week, mean (CI: 95%) | 3.1 (1.5;4.8) | 5.1 (3.2;7.1) |
| None, No. (%) | 8 (38) | 1 (5) |

Abbreviations: CI, confidence interval; BCS, breast cancer survivors; CON, controls.
